# 基于超高效液相色谱-四极杆-静电场轨道阱高分辨质谱的刺五加注射液化学成分分析

**DOI:** 10.3724/SP.J.1123.2022.06005

**Published:** 2023-03-08

**Authors:** Wenyi YU, Huimin WU, Xiujie GUO, Shumei YAN, Xiangjie LIU, Zhujun WANG, Chaoran WANG, Aijin SHEN, Xinmiao LIANG

**Affiliations:** 1.中国科学院大连化学物理研究所, 中国科学院分离分析化学重点实验室, 辽宁 大连 116023; 1. CAS Key Laboratory of Separation Science for Analytical Chemistry, Dalian Institute of Chemical Physics, Chinese Academy of Sciences, Dalian 116023, China; 2.中科院大化所中国医药城生物医药创新研究院, 江苏 泰州 225300; 2. DICP-CMC Innovation Institute of Medicine, Taizhou 225300, China; 3.江西省中药药效物质基础重点实验室, 赣江中药创新中心, 江西 南昌 330000; 3. Jiangxi Provincial Key Laboratory for Pharmacodynamic Material Basis of Traditional Chinese Medicine, Ganjiang Chinese Medicine Innovation Center, Nanchang 330000, China; 4.黑龙江乌苏里江制药有限公司, 黑龙江 虎林 158417; 4. Heilongjiang Wusuli River Pharmaceutical Co., Ltd., Hulin 158417, China

**Keywords:** 超高效液相色谱-四极杆-静电场轨道阱高分辨质谱, 苯丙素类, 木脂素类, 香豆素类, 有机酸类, 刺五加注射液, ultra-high performance liquid chromatography-quadrupole-electrostatic field orbitrap high-resolution mass spectrometry (UHPLC-Q/Orbitrap HRMS), phenylpropanoids, lignans, coumarins, organic acids, Ciwujia injection

## Abstract

刺五加注射液是临床治疗脑血管疾病及中枢神经系统疾病的常用药物,能明显改善急性脑梗死患者血脂水平及内皮细胞功能,促进缺血脑组织神经干细胞的增殖,对高血压、脑梗死等脑血管疾病具有良好的疗效。目前刺五加注射液的药效物质基础研究还比较薄弱,制约了其临床作用机理的深入研究。该研究基于超高效液相色谱-四极杆-静电场轨道阱高分辨质谱联用(UHPLC-Q/Orbitrap HRMS)技术对刺五加注射液的化学成分进行定性分析,采用BEH Shield RP18色谱柱(100 mm×2.1 mm, 1.7 μm),以0.1%甲酸水溶液和乙腈为流动相进行梯度洗脱,流速为0.4 mL/min,柱温30 ℃,在加热电喷雾离子源正、负离子两种模式下采集刺五加注射液的一级、二级质谱数据。通过调研文献收集刺五加已报道成分的名称、分子式和结构式,建立刺五加化学成分列表用于数据后处理,根据高分辨质谱提供的精确质量数和碎片离子信息,结合对照品比对、数据库匹配及裂解规律分析,从刺五加注射液中鉴定出102个化合物,包括62个苯丙素类、23个有机酸类、7个核苷类、1个环烯醚萜类和9个其他类成分,其中有65个成分为从刺五加注射液中首次鉴定得到。该研究建立了刺五加注射液的UHPLC-Q/Orbitrap HRMS分析方法,可以全面、快速地分析刺五加注射液的化学成分,新发现的27个苯丙素类成分为刺五加注射液临床治疗神经系统疾病提供了一定的化学成分依据,也为其药效作用机理的深入阐明提供了新的研究目标。

## Abstract (en)

Ciwujia injection is commonly used to treat cerebrovascular and central nervous system diseases in clinical practice. It can significantly improve blood lipid levels and endothelial cell function in patients with acute cerebral infarction and promote the proliferation of neural stem cells in cerebral ischemic brain tissues. The injection has also been reported to have good curative effects on cerebrovascular diseases, such as hypertension and cerebral infarction. At present, the material basis of Ciwujia injection remains incompletely understood, and only two studies have reported dozens of components, which were determined using high performance liquid chromatography-quadrupole time-of-flight mass spectrometry (HPLC-Q-TOF MS). Unfortunately, the lack of research on this injection restricts the in-depth study of its therapeutic mechanism.In the present study, a qualitative method based on ultra-high performance liquid chromatography-quadrupole-electrostatic field orbitrap high-resolution mass spectrometry (UHPLC-Q/Orbitrap HRMS) was developed to analyze the chemical components of Ciwujia injection. Separation was performed on a BEH Shield RP18 column (100 mm×2.1 mm, 1.7 μm) using 0.1% formic acid aqueous solution (A) and acetonitrile (B) as the mobile phases, and gradient elution was performed as follows: 0-2 min, 0%B; 2-4 min, 0%B-5%B; 4-15 min, 5%B-20%B; 15-15.1 min, 20%B-90%B; 15.1-17 min, 90%B. The flow rate and column temperature were set to 0.4 mL/min and 30 ℃ respectively. MS^1^ and MS^2^ data were acquired in both positive- and negative-ion modes using a mass spectrometer equipped with an HESI source. For data post-processing, a self-built library including component names, molecular formulas, and chemical structures was established by collecting information on the isolated chemical compounds of *Acanthopanax senticosus*. The chemical components of the injection were identified by comparison with standard compounds or MS^2^ data in commercial databases or literature based on precise relative molecular mass and fragment ion information. The fragmentation patterns were also considered. For example, the MS^2^ data of 3-caffeoylquinic acid (chlorogenic acid), 4-caffeoylquinic acid (cryptochlorogenic acid), and 5-caffeoylquinic acid (neochlorogenic acid) were first analyzed. The results indicated that these compounds possessed similar fragmentation behaviors, yielding product ions at *m/z* 173 and *m/z* 179 simultaneously. However, the abundance of the product ion at *m/z* 173 was much higher in 4-caffeoylquinic acid than in 5-caffeoylquinic acid or 3-caffeoylquinic acid, and the fragment signal at *m/z* 179 was much stronger for 5-caffeoylquinic acid than for 3-caffeoylquinic acid. Four caffeoylquinic acids were identified using a combination of abundance information and retention times. MS^2^ data in commercial database and literature were also used to identify unknown constituents. For example, compound 88 was successfully identified as possessing a relative molecular mass and neutral losses similar to those of sinapaldehyde using the database, and compound 80 was identified as salvadoraside because its molecular and fragmentation behaviors were consistent with those reported in the literature. A total of 102 constituents, including 62 phenylpropanoids, 23 organic acids, 7 nucleosides, 1 iridoid, and 9 other compounds, were identified. The phenylpropanoids can be further classified as phenylpropionic acids, phenylpropanols, benzenepropanals, coumarins, and lignans. Among the detected compounds, 16 compounds were confirmed using reference compounds and 65 compounds were identified in Ciwujia injection for the first time. This study is the first to report the feasibility of using the UHPLC-Q/Orbitrap HRMS method to quickly and comprehensively analyze the chemical components of Ciwujia injection. The 27 newly discovered phenylpropanoids provide further material basis for the clinical treatment of neurological diseases and new research targets for the in-depth elucidation of the pharmacodynamic mechanism of Ciwujia injection and its related preparations.

脑血管病是目前我国患病率、致残率、病死率最高的疾病之一,严重威胁着人们的生命健康安全^[1,2]^,根据《中国卒中报告2019》, 2018年中国居民脑血管病死亡率为149.49/10万,死亡人数约157万,占当年总死亡人数的22.33%^[3]^。刺五加注射液是五加科植物刺五加*Acanthopanax senticosus* (Rupr. et Maxim.) Harms的干燥根和根茎或茎经过提取、过滤和灭菌等工艺制得的注射液。研究表明刺五加注射液能明显改善急性脑梗死患者的血脂水平及内皮细胞功能,促进缺血脑组织神经干细胞的增殖,对高血压、脑梗死等脑血管疾病具有良好的疗效^[4,5]^。除此之外,刺五加注射液对中枢神经系统有良好的镇静作用^[5]^,可以改善焦虑、烦躁、失眠等症状,临床上常用于治疗情志异常类疾病^[6]^和神经系统疾病^[7]^。

中药物质基础研究是药效及作用机理研究的前提,目前针对刺五加药材化学成分的研究已有较多报道^[8⇓⇓-11]^,而针对刺五加注射液化学成分的研究却十分有限,仅有Xie等^[12]^采用高效液相色谱-四极杆飞行时间质谱技术(HPLC-Q-TOF-MS)从刺五加注射液中鉴定出22个化合物,黄婧等^[13]^采用HPLC-Q-TOF-MS从刺五加注射液中鉴定出54个苯丙素类成分。作为刺五加临床用药的主要形式,刺五加注射液化学成分研究对明确刺五加药材及多种刺五加制剂的药效物质基础非常重要。

静电场轨道阱是近年来发展起来的一种新型质谱质量分析器,相比于飞行时间质谱具有扫描速度更快、分辨率更高等优势,尤其是与超高效液相色谱(UHPLC)结合后可大大缩短样品分析时间,提高化学成分的鉴定效率,近年来已成为复杂样品分析的有力工具,广泛用于各种中药药效物质基础研究^[14]^。采用超高效液相色谱-四极杆-静电场轨道阱高分辨质谱联用(UHPLC-Q/Orbitrap HRMS)技术,建立刺五加注射液的快速分析方法,深入研究其化学成分,进一步明确其物质基础,对于深入阐明刺五加及其相关制剂的药效作用机理具有重要的意义。

## 1 实验部分

### 1.1 仪器、试剂与材料

Vanquish UHPLC超高效液相色谱仪(Thermo Scientific,美国),配有自动进样器、二元梯度泵、柱温箱和二极管阵列检测器;Q Exactive Plus超高分辨率四极杆-静电场轨道阱质谱仪(Thermo Scientific,美国),配有加热电喷雾离子(HESI)源、Xcalibur化学工作站和Compound Discoverer 3.2数据处理软件;Milli-Q超纯水制备仪(Milli-pore,美国); XSR105 DualRange十万分之一电子天平(METTLER TOLEDO,瑞士); SORVALL ST8台式离心机(Thermo Scientific,美国); KQ5200DE型数控超声波清洗器(昆山市超声仪器有限公司)。

刺五加提取液(批号21080201-01)和注射液(批号202008012)由黑龙江乌苏里江制药有限公司提供,提取液为药材经过纯水提取、减压浓缩、醇沉等步骤制得,提取液再经过滤、稀释后制得注射液,制备过程中未添加其他辅料。标准品信息见表1。

**表1:** 对照品信息及在混合标准溶液中的质量浓度

Compound	Chinese name	Lot number	Purity	Manufacturer	Mass concentration/ (mg/L)
Adenosine	腺苷	DSTDX004701	≥99%	Chengdu DeSiTe Biological	20.00
L-Phenylalanine	L-苯丙氨酸	DSTDB013601	≥99%	Technology Co. Ltd. (China)	41.20
Eleutheroside B1	刺五加苷B1	DST211011-139	≥98%		41.40
Azelaic acid	壬二酸	DST200825-088	≥99%		37.80
Neochlorogenic acid	新绿原酸	BP0083	≥98%	Biopurify Phytochemicals	2.86
Cryptochlorogenic acid	隐绿原酸	24A007	≥98%	Ltd. (China)	2.44
1,3-Dicaffeoylquinic acid	1,3-二咖啡酰奎宁酸	BP0005	≥98%		2.94
Protocatechualdehyde	原儿茶醛	PRF8060647	≥99%		3.42
Puerarin	葛根素	BP1176	>98%		26.60
Guanosine	鸟苷	0-8119	≥98%	Chengdu Push Bio-technology	2.44
5-Hydroxymethylfurfural	5-羟甲基糠醛	BP3035	≥97%	Co. Ltd. (China)	63.80
Protocatechuic acid	原儿茶酸	PS0619	≥98%		27.00
Syringin	紫丁香苷	BP0525	≥99%		2.96
Chlorogenic acid	绿原酸	PS000627	≥98%		2.40
Caffeic acid	咖啡酸	YY90096	≥98%		2.86
Eleutheroside E	刺五加苷E	BP0527	≥99%		2.48
Isofraxidin	异嗪皮啶	BP0783	≥97%		2.84
3'-Methoxypuerarin	3'-甲氧基葛根素	PS011422	>98%		48.00

### 1.2 溶液制备

对照品溶液:称定各对照品适量,分别用70%(v/v)甲醇水溶液溶解,得到对照品储备液,置于4 ℃防爆冰箱中保存,质量浓度为0.2~0.7 mg/mL。取对照品储备液适量,配制成混合标准溶液,混合标准溶液中各化合物浓度见表1。

供试品溶液:取刺五加注射液100 μL,加入900 μL纯水稀释10倍后离心(8000 r/min, 10 min),取上清液,即得供试品溶液。

### 1.3 仪器条件

色谱条件:Waters ACQUITY UPLC BEH Shield RP18色谱柱(100 mm×2.1 mm, 1.7 μm);流动相A为0.1%甲酸水溶液,流动相B为乙腈;梯度洗脱程序:0~2 min, 0%B; 2~4 min, 0%B~5%B; 4~15 min, 5%B~20%B; 15~15.1 min, 20%B~90%B; 15.1~17 min, 90%B。流速:0.4 mL/min;柱温:30 ℃;进样量:2 μL;检测波长:280 nm。

质谱条件:HESI源,正、负离子模式下分别进行检测;屏蔽气流速:45 arb;辅助气流速:10 arb;正离子模式喷雾电压:3.5 kV,负离子模式喷雾电压:3.0 kV;离子传输管温度:380 ℃; S-lens RF水平: 50;干燥气温度:400 ℃;数据采集模式:一级质谱全扫描加数据依赖的二级质谱扫描(Full MS/dd-MS^2^);质量扫描范围:100~1300;分辨率:70000(Full MS), 17500(MS^2^);碰撞能(stepped NCE): 10、30和50; TopN: 3;动态排除:2 s。

### 1.4 数据处理

原始数据采用Xcalibur软件和Compound Discoverer 3.2(CD)软件进行处理,扣除背景空白和提取色谱峰后,结合文献报道的刺五加药材及刺五加注射液的化学成分^[9,13,15⇓-17]^,首先进行一级精确相对分子质量的匹配,然后采用CD软件在mzCloud和mzVault数据库中进行二级质谱图匹配,对于匹配分数≥75的成分,人工确认其二级谱图的匹配情况,对于没有匹配分数的成分,结合文献报道的碎片和裂解规律进行鉴定,最后采用标准品对部分化合物进行确认。

## 2 结果与讨论

采用UHPLC-Q/Orbitrap HRMS分析刺五加提取液和注射液,刺五加注射液的紫外色谱图和正、负离子模式总离子流图见图1,根据1.4节的数据处理方法从中鉴定出102个化学成分,包括62个苯丙素类、23个有机酸类、7个核苷类、1个环烯醚萜类和9个其他类成分,其中有65个成分为从刺五加注射液中首次鉴定得到,包括27个苯丙素类、22个有机酸类、7个核苷类、1个环烯醚萜类和8个其他类,并系统归纳总结了各类型化合物的裂解规律。

**图1:**
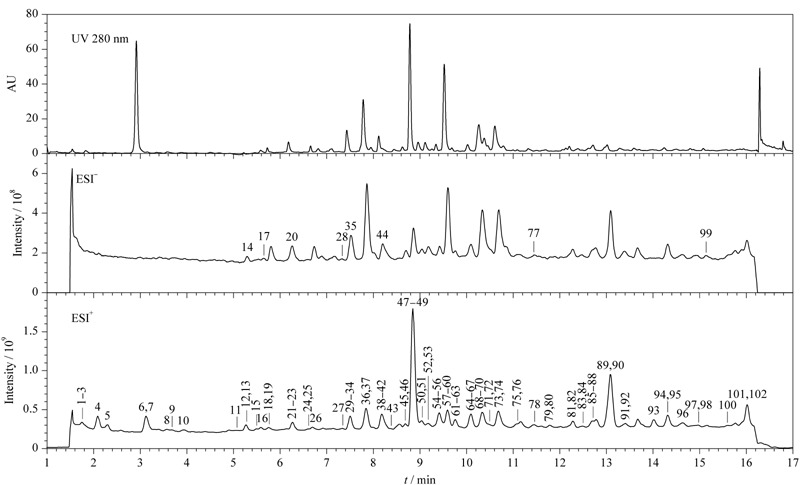
刺五加注射液的紫外色谱图和总离子流色谱图

### 2.1 刺五加注射液中苯丙素类成分的鉴定

苯丙素类化合物是指一类以C_6_-C_3_为基本单元的化合物。在植物体内,这种单元可独立形成化合物,如苯丙醇类化合物、苯丙醛类化合物和苯丙酸类化合物,也可以2个、3个甚至多个单元聚合形成某一类化合物,如木脂素类化合物,还可以形成多种氧化程度不同的衍生物,如香豆素类化合物。刺五加注射液中包含众多不同结构类型的苯丙素类化合物。

#### 2.1.1 苯丙酸类成分的鉴定

苯丙酸类成分在植物中常与不同的醇、氨基酸、糖、有机酸结合形成糖苷类或酯类化合物,且易发生糖苷键和酯键的断裂,丢失葡萄糖(-162 Da)、咖啡酰基(-162 Da)、阿魏酰基(-176 Da)、香豆酰基(-146 Da)等中性基团,或者丢失其他基团产生咖啡酸(*m/z* 179)、阿魏酸(*m/z* 193)、香豆酸(*m/z* 163)等碎片离子。刺五加注射液中有很多奎宁酸与咖啡酰基、阿魏酰基和苯丙酰基结合而成的异构体,本实验通过标准品研究了这类异构体的裂解规律:隐绿原酸(cryptochlorogenic acid,化合物70)相比于新绿原酸(neochlorogenic acid,化合物37)和绿原酸(chlorogenic acid,化合物73)具有更高丰度的*m/z* 173碎片,而新绿原酸相比于绿原酸具有更高丰度的*m/z* 179碎片,这一现象与文献^[18,19]^报道一致,图2以绿原酸为例描述了这类化合物的裂解途径。值得一提的是,绿原酸和隐绿原酸的出峰顺序与文献报道略有差异,可能是因为这两个成分的保留时间比较接近,在不同类型反相色谱柱上的选择性有差异导致的。利用以上碎片丰度差异以及化合物在BEH Shield RP18色谱柱上的出峰顺序对苯丙酰基奎宁酸类化合物(9、10、15、16、19)和阿魏酰基奎宁酸类化合物(31、50、53)进行了鉴定。

**图2:**
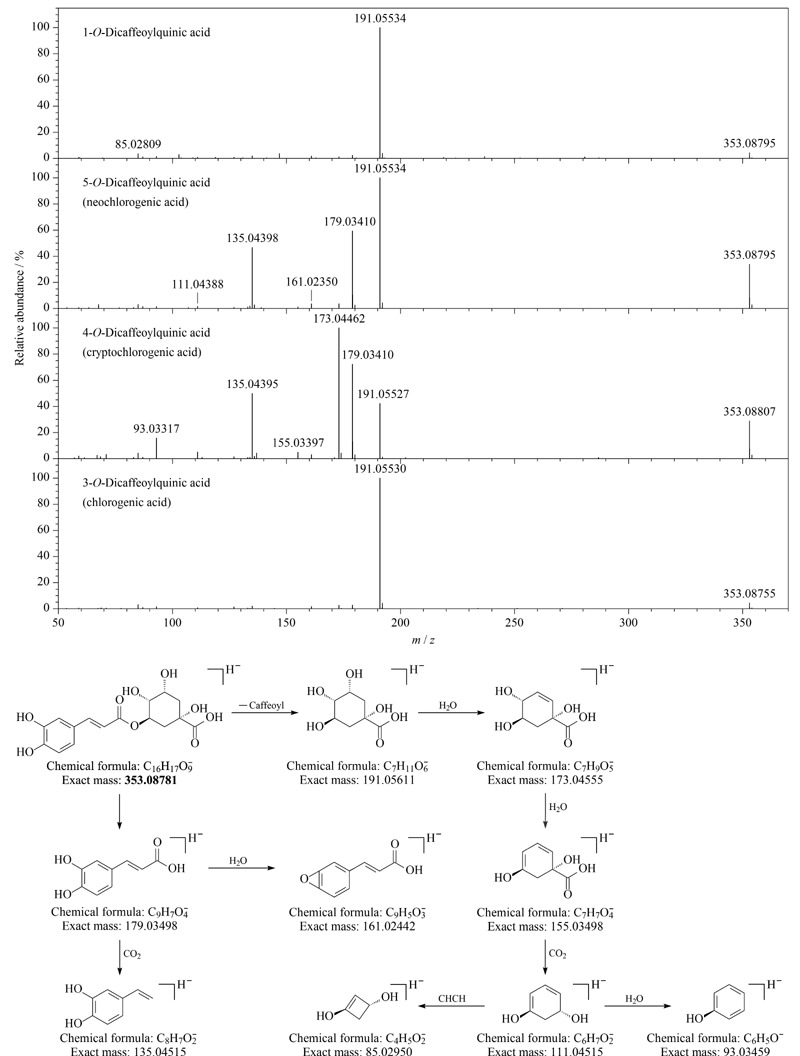
咖啡酰奎宁酸类化合物的二级质谱图和绿原酸的质谱裂解途径

除上述已报道的成分外,从刺五加注射液中首次鉴定了10个苯丙酸类成分,具体鉴定过程如下:化合物30、52、59、67、71具有相同的相对分子质量和二级碎片,分子式为C_10_H_10_O_4_,主要碎片*m/z* 177和*m/z* 163为丢失H_2_O和CH_3_OH产生,经CD软件匹配二级谱图将其鉴定为3-羟基-5-甲氧基-肉桂酸(3-hydroxy-5-methoxy-cinnamic acid)及其在不同取代位置的异构体;化合物82和85互为同分异构体,其质量数和二级碎片与刺五加果实中报道的(2*E*)-3-[4-({(1*S*,2*S*)-1-[4-(*β*-D-吡喃葡萄糖基氧基)-3-甲氧基苯基]-1,3-二羟基-2-丙烯基}氧基)-3-甲氧基苯基]丙烯酸((2*E*)-3-[4-({(1*S*,2*S*)-1-[4-(*β*-D-glucopyranosyloxy)-3-methoxyphenyl]-1,3-dihydroxy-2-propanyl}oxy)-3-methoxyphenyl] acrylic acid)一致^[17]^,该化合物在刺五加根和根茎中未见报道;化合物84的分子式为C_32_H_36_O_17_,碎片*m/z* 529为[M-Glu]^-^,碎片*m/z* 353和*m/z* 191为继续丢失阿魏酰基和咖啡酰基产生,结合文献报道将其鉴定为阿魏酰咖啡酰奎宁酸葡萄糖苷(feruloyl caffeicyl quinic acid glucoside)^[9]^;化合物41在正离子模式下有碎片*m/z* 131和*m/z* 103,为丢失CH_3_OH和CO产生,经CD软件匹配二级谱图将其鉴定为肉桂酸甲酯(methyl cinnamate);化合物64先丢失H_2_O产生*m/z* 147,再丢失CO和·CH_3_产生*m/z* 119和*m/z* 103,经CD软件匹配二级谱图将其鉴定为D(+)-苯乳酸(D(+)-phenyllactic acid)。

#### 2.1.2 苯丙醇类成分的鉴定

从刺五加注射液中鉴定到3个苯丙醇类成分,分别为紫丁香苷葡萄糖苷(syringinoside,化合物39)、松柏苷(coniferin,化合物42)和紫丁香苷(syringin,化合物48),其中紫丁香苷葡萄糖苷和松柏苷为首次从刺五加注射液中鉴定得到,其在质谱中的裂解从糖苷键的断裂开始,然后发生H_2_O、·CH_3_等中性丢失,松柏苷的裂解途径见图3。

**图3:**
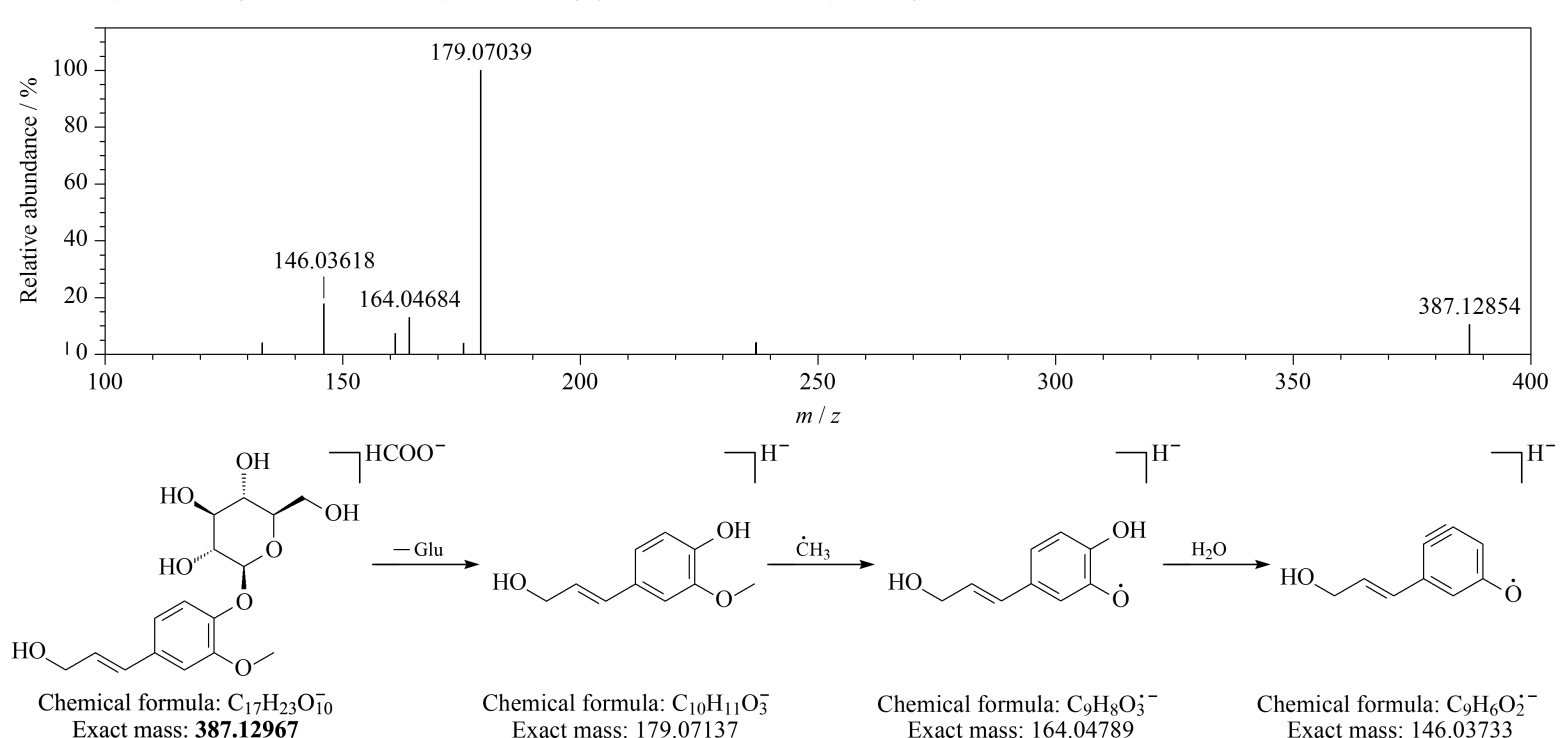
松柏苷的二级质谱图和质谱裂解途径

#### 2.1.3 苯丙醛类成分的鉴定

从刺五加注射液中鉴定出1个苯丙醛类成分芥子醛(sinapaldehyde,化合物88),该化合物在正离子模式下响应较好,在负离子模式下响应较弱,质谱裂解以中性丢失CO、CH_3_OH、·CH_3_等为主,经CD软件匹配二级谱图将其鉴定为芥子醛,其裂解途径见图4,该化合物为首次从刺五加中鉴定得到。

**图4:**
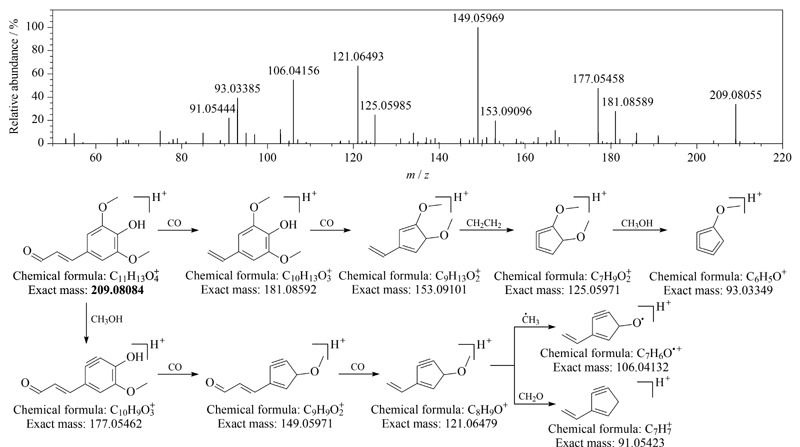
芥子醛的二级质谱图和质谱裂解途径

#### 2.1.4 香豆素类成分的鉴定

从刺五加注射液中鉴定到9个香豆素类成分,均为简单香豆素及其与葡萄糖、鼠李糖连接形成的糖苷类化合物。化合物55、62、66、90为同分异构体,正、负离子模式加合峰和二级碎片均相同,负离子模式下碎片离子*m/z* 206和*m/z* 191为分子离子峰连续丢失·CH_3_产生,继续连续丢失CO产生*m/z* 163、*m/z* 135和*m/z* 107,化合物90通过标准品鉴定为异嗪皮啶(isofraxidin),其裂解途径见图5,其余3个化合物鉴定为秦皮素定(fraxidin)或白蜡树精(fraxinol)或网状菌醇(reticulol),为异嗪皮啶不同取代基位置差异的异构体,这3个异构体为首次从刺五加注射液中鉴定得到。

**图5:**
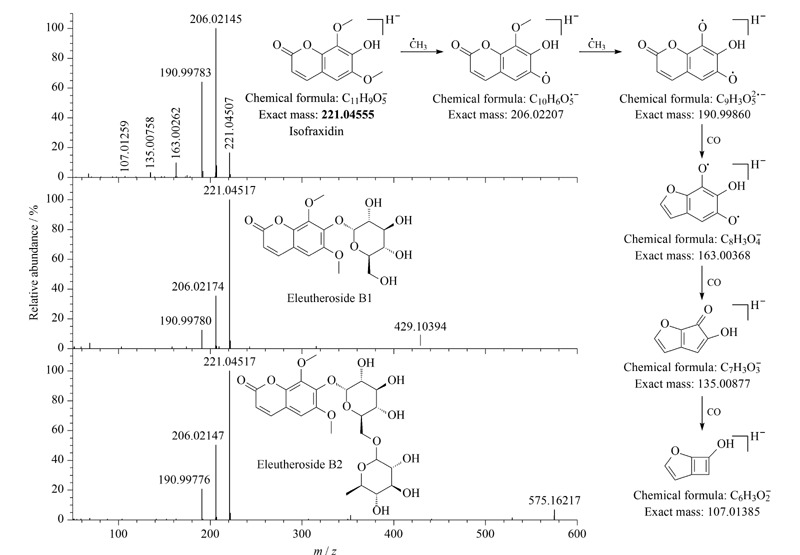
异嗪皮啶、刺五加苷 B1、刺五加苷 B2的二级质谱图和异嗪皮啶的质谱裂解途径

化合物56和化合物63根据其正、负离子模式加合峰推测其分子式分别为C_17_H_20_O_10_和C_23_H_30_O_14_,二级碎片*m/z* 221分别为[M-Glu]^-^和[M-Glu-Rha]^-^,其余碎片离子与化合物90一致,根据标准品对比将化合物56鉴定为刺五加苷B1(eleutheroside B1),根据文献^[13]^推测化合物63为刺五加苷B2(eleutheroside B2),其二级质谱图见图5。化合物69与化合物56为同分异构体,其丢失葡萄糖之后的碎片与秦皮素定和异嗪皮啶等化合物相同,将其鉴定为秦皮素定葡萄糖苷(fraxidin-*O*-glucoside)。

化合物46和57通过一级相对分子质量匹配分别得到化合物东莨菪素(scopoletin)和秦皮素(fraxetin),二者相差一个羟基,其质谱裂解主要为丢失·CH_3_、H_2_O、CO、CH_3_OH等中性基团,符合香豆素类化合物的一般裂解规律,此外经CD软件匹配二级谱图发现,这两个化合物的二级谱图与数据库中标准品的谱图匹配良好,因此分别将化合物46和57鉴定为东莨菪素和秦皮素,二者均为首次从刺五加注射液中鉴定得到,其裂解途径见图6。

**图6:**
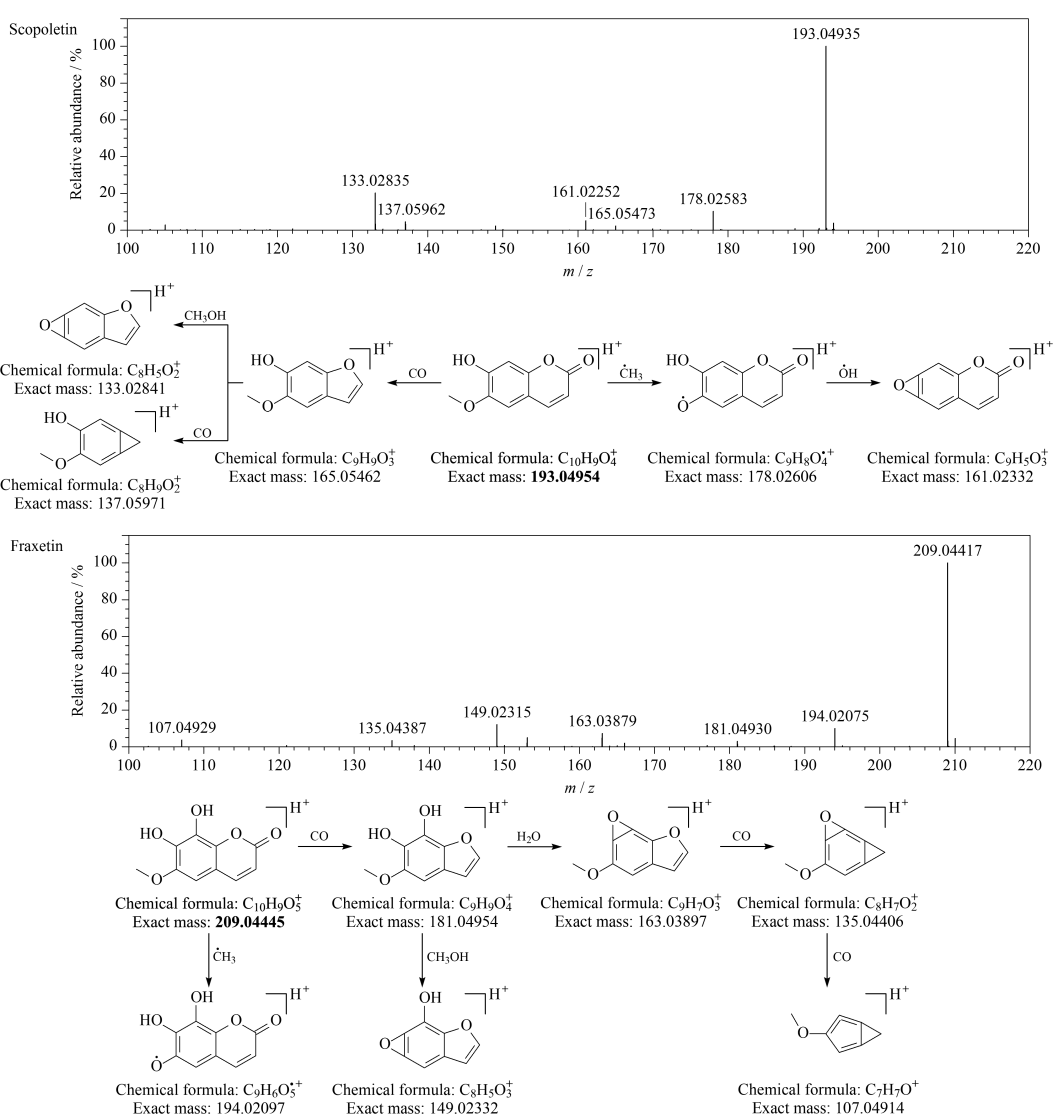
东莨菪素和秦皮素的二级质谱图和质谱裂解途径

#### 2.1.5 木脂素类成分的鉴定

刺五加中的木脂素通常以单糖苷或双糖苷的形式存在^[20⇓-22]^,一般具有相对较高的相对分子质量(>500)。从刺五加注射液中鉴定出16个木脂素类成分,包括6种结构类型:双环氧木脂素类(83、87、91、100、102)、氧新木脂素类(76、78、79)、苯骈呋喃类(77、89、95)、单环氧木脂素类(80、96、97)、芳基萘类(75)和二芳基丁烷类(93)。此类化合物的二级质谱通常会先丢失糖基产生苷元的碎片离子,然后根据苷元上取代基的不同发生·CH_3_、CH_2_O、CH_3_OH、·OCH_3_等中性丢失,进一步碰撞还会产生苷元中醚键或呋喃环等结构的断裂,生成更小的二级碎片。如图7阿拉克苷(salvadoraside)的裂解途径,准分子离子峰*m/z* 789连续丢失糖基生成*m/z* 581和*m/z* 419,碎片*m/z* 419会丢失CH_2_O或发生四氢呋喃环裂解生成*m/z* 389、*m/z* 222等碎片,该化合物此前只在刺五加茎中有报道^[23]^,本工作为首次从刺五加注射液中鉴定得到。

**图7:**
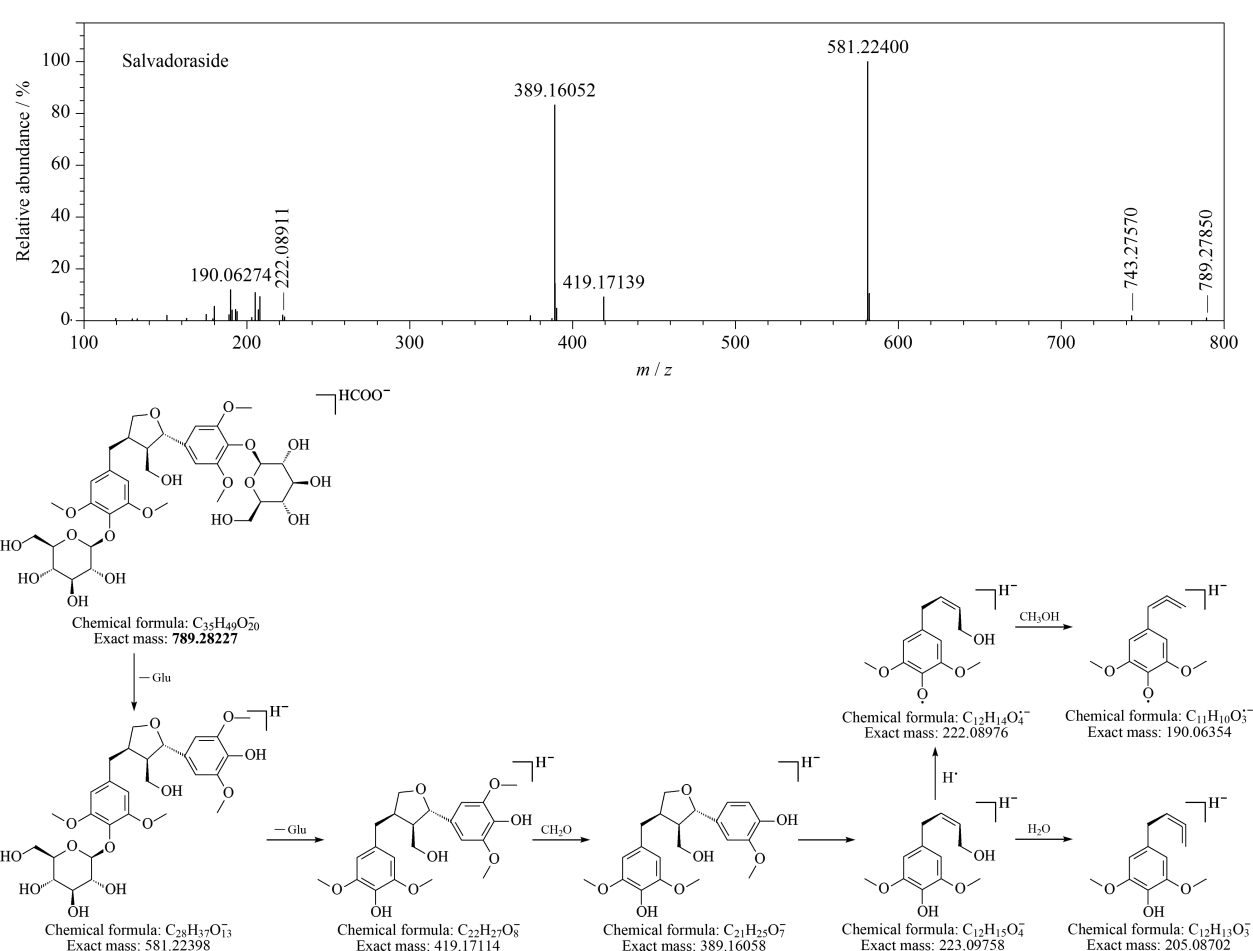
Salvadoraside的二级质谱图和质谱裂解途径

根据文献报道,刺五加中的苯丙素类成分具有保护神经元、改善植物神经紊乱、调节免疫力、缓解焦虑等多种药理活性^[24⇓⇓⇓-28]^。刺五加苷B、刺五加苷E和异嗪皮啶是刺五加中的主要苯丙素类成分,这3个成分在μmol/L水平对*β*淀粉样蛋白诱导的轴突和树突萎缩具有明显的保护作用,而轴突和树突萎缩是导致阿尔茨海默症记忆缺失的直接原因^[24]^。此外,刺五加苷B还具有心血管保护作用和抗肿瘤作用,刺五加苷E还具有免疫调节作用、抗氧化作用和降血糖作用等^[28]^。本研究从刺五加注射液中鉴定出包含上述3个主要成分在内的62个苯丙素类成分,其中27个为首次鉴定到,这些成分为刺五加注射液进一步的药理研究奠定了基础。

### 2.2 刺五加注射液中有机酸类成分的鉴定

除了苯丙素类成分之外,刺五加注射液中还包含大量小分子有机酸类成分,如水杨酸(salicylic acid)、原儿茶酸(protocatechuic acid)、壬二酸(azelaic acid)等,这些化合物多数为苯环或长链烷烃连接羟基或羧基而成,一般在负离子模式下响应较好^[29]^,二级质谱以丢失H_2_O、CO、CO_2_、HCOOH等小分子为主,从刺五加注射液中鉴定出23个有机酸类成分。

综上,刺五加注射液的化学成分鉴定结果见表2。

**表2:** 刺五加注射液的化学成分鉴定结果

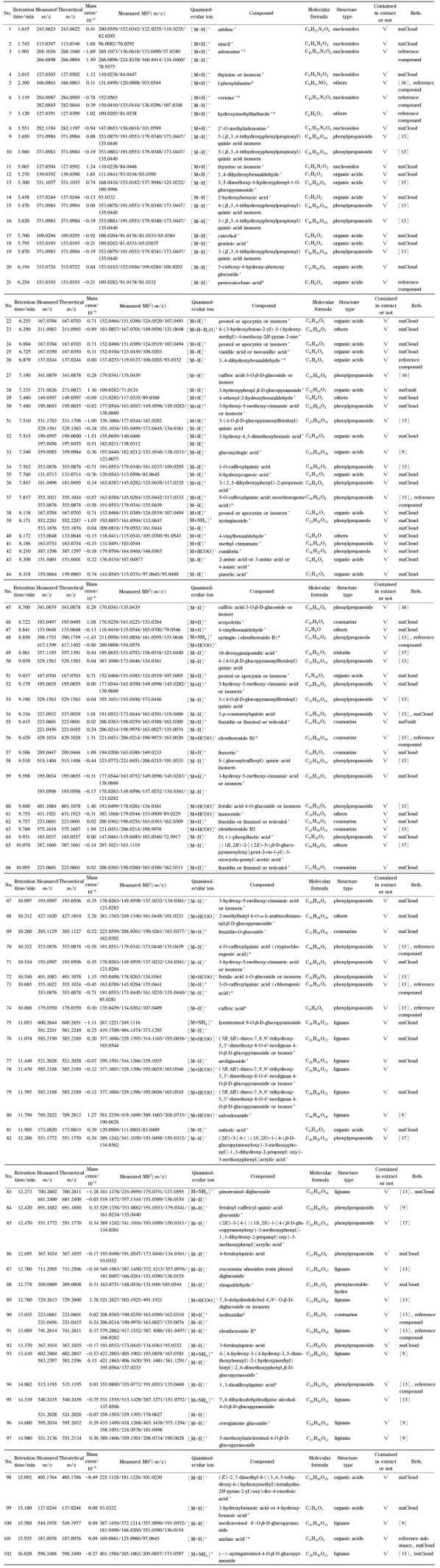

* Compound identified from Ciwujia injection for the first time; a: Compound identified by comparison with reference compound.

### 2.3 刺五加注射液中黄酮类成分测定

鉴于刺五加注射液现行质量标准WS_3_-B-3425-98^[30]^以紫外-可见分光光度法测定总黄酮的含量作为主要质量控制方法,但上述质谱鉴定发现主成分均非黄酮类成分,因此我们对刺五加注射液的黄酮类成分进行了探索。考虑到没有文献报道刺五加注射液中的黄酮类成分,我们参考刺五加药材化学成分的研究工作^[8]^,在刺五加注射液中提取相应的黄酮类成分的提取离子流图(EIC),除葛根素和3'-甲氧基葛根素(见图8)可以提到响应很低的色谱峰之外(用标准品对这两个成分进行确认,发现保留时间基本一致,但是由于这两个成分在刺五加注射液中的含量非常低,质谱响应在10^4^水平,即使采用target模式也无法采集到有效的二级谱图,因此无法对其碎片进行进一步确认),其他黄酮类成分(包括芦丁、金丝桃苷、山柰酚、槲皮苷、槲皮素、金合欢素、大豆苷、3'-甲氧基大豆苷、4'-甲氧基葛根素)提取不到色谱峰。我们推测是由于黄酮类成分整体极性相对较弱,注射液采用水提工艺无法有效提取。此外,文献^[31]^报道芦丁等黄酮类成分主要存在于刺五加叶中,因此也可能是文献所用的刺五加药材中混入了刺五加叶。

**图8:**
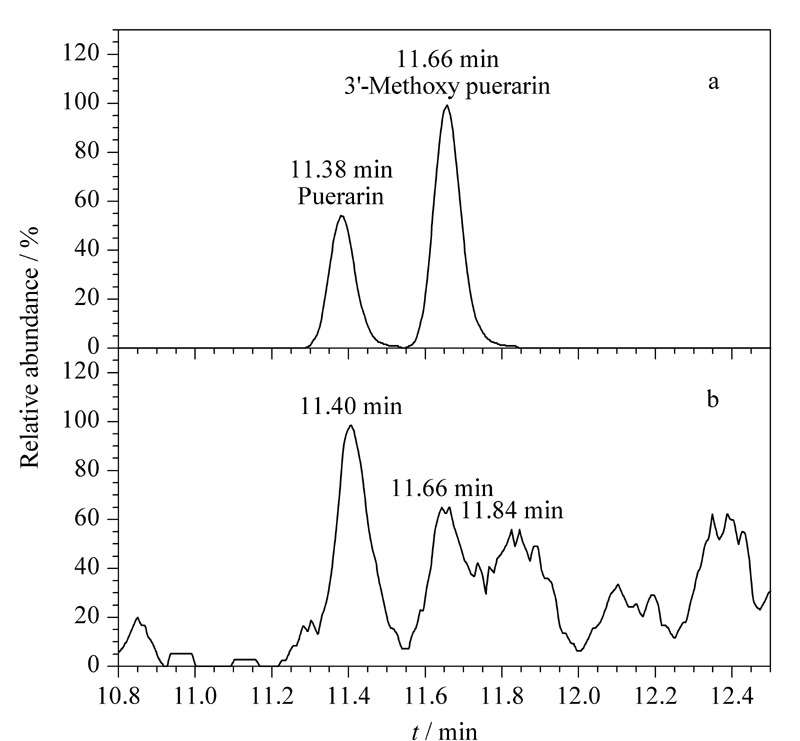
葛根素和3'-甲氧基葛根素在(a)标准溶液和(b)刺五加注射液中的提取离子色谱图

## 3 结论

本研究通过建立UHPLC-Q/Orbitrap HRMS分析方法,从刺五加注射液中共鉴定出102个化合物,其中包含62个苯丙素类成分,为刺五加注射液临床治疗神经系统疾病和心血管疾病提供了一定的化学成分依据。除此之外,本研究首次鉴定了27个苯丙素类成分、22个有机酸类成分、7个核苷类成分、1个环烯醚萜类成分和8个其他类成分,丰富了刺五加注射液的物质组成信息,这些新成分的发现为刺五加注射液作用机制的阐明奠定了科学数据基础。与此同时,我们针对黄酮类成分进行了探索,发现黄酮类成分并非刺五加注射液的主要成分,现有质量标准通过测定总黄酮的含量可能并不能对注射液进行有效的质量控制。除上述化学成分类型之外,有研究报道刺五加中还含有三萜皂苷类成分,该类成分与人参皂苷类成分药理活性较为相似,与刺五加对心脑血管的治疗作用直接相关^[20]^,然而在本次实验中从刺五加注射液中未鉴定到三萜皂苷类成分,在对刺五加提取液进行鉴定时也未发现三萜皂苷类成分,推测可能是由于三萜皂苷类成分极性较弱,在水中溶解度较差导致的,因此现有刺五加注射液的制备方法可能还存在进一步改进的空间。
